# Potentially traumatic experiences of seafarers

**DOI:** 10.1186/s12995-019-0238-9

**Published:** 2019-05-31

**Authors:** Hans-Joachim Jensen, Marcus Oldenburg

**Affiliations:** 1 University of Applied Sciences, Flensburg, Germany; 2 Institute for Occupational and Maritime Medicine Hamburg (ZfAM), University Medical Center Hamburg-Eppendorf (UKE), Seewartenstrasse 10, 20459 Hamburg, Germany

**Keywords:** Maritime, Vessel, Trauma, Seafarer

## Abstract

**Background:**

The aim of the present study is to assess the extent to which seafarers had been affected by severe mental stress situations and what possible psychological effects they may have.

**Methods:**

During the voyages of 22 German ships, a psychologically trained investigator interviewed 323 seafarers about severe mental stress or potentially traumatic events on board (participation rate 88.5%). Furthermore, a psychologist conducted semi-standardized interviews in 12 shipping companies and in 8 stations of the German Seafarers’ Mission on seafarers’ traumatic experiences.

**Results:**

Of the seafarers surveyed on board, 116 (35.9%) experienced ship wrecks/severe accidents and 55 (17.0%) piracy on board. Experiences with stowaways were indicated by 126 seafarers (39.0%). Repeatedly having to unintentionally think about these events, being reminded of them by certain noises, smells, etc., or often dreaming of them were after-effects in 97 (83.6%) of the affected seafarers after ship wrecks/serious personal accidents on board and in 42 crew members (76.4%) after threats of piracy. Experiences of threat from stowaways had particularly affected non-European seafarers or ratings.

According to the interviews with the shipping companies, a total of 14 deaths occurred in the last 3 years in the 12 interviewed shipping companies (due to heart attacks (3), severe cancer (3), very serious accidents (3), suicides (2) and 3 with unknown causes of death). In relation to the container ships of the investigated shipping companies, these frequencies correspond to a mortality rate of 78.4 per 100,000 seafarer years.

According to the interviews in 8 Seafarers’ Missions, these organizations are in charge of emergency counselling in the form of crisis intervention when a seafarer has experienced serious psychological stress.

**Discussion:**

A serious injury or even the death of a member is a serious psychological stress experience for the other crew members in the closed social system on board. These events are particularly distressing for Filipino seafarers with their strong sociocentric bonds and religious values. Priestly support is often required in order to stabilize the Filipino crew members.

**Conclusion:**

As a preventive measure, psychoeducation for seafarers should be established for coping with extreme mental stress situations.

## Background

Within a small crew working together and living in a closed social system over a longer period of time, a serious injury or even the death of one crew member could cause a secondary mental trauma for the rest of the crew [1]. Such traumatization is even more likely if an unaffected crew member empathizes with the primary victim and, at the same time, experiences being unable to help [2]. The confrontation with the most seriously injured, with severely bleeding wounds, and unsuccessful rescue and resuscitation attempts can be long-lasting experiences [3].

In shipping, severe psychological stress or potentially traumatic events can result from ship wrecks and accidents, threats and experiences of violence from piracy, stowaways and in life-threatening rescue situations during the rescue of refugees on the high seas [4, 5].

An own, so far unpublished analysis of sea accidents on ships in German territory revealed casualties on board 20 container ships in the period from 2005 to 2014. An investigation by the Federal Bureau of Maritime Casualty Investigation (BSU) (Maritime Safety Investigation -Law) identified accidents in which crew members were seriously or fatally injured, went overboard, experienced the total loss of their ship or were also responsible for it, as the major causes. However, there are also fatalities due to “intentional self-harm and assault” on board. In the study by Grappasonni et al. (2012) [6], 14.3% of observed external causes of seafarers’ mortality over a period of 25 years were related to this kind of fatality (9 of a total of 64 crew members).

The aim of the present study is to assess the frequency and the sustained shaping of severe stress experiences among seafarers. In addition, it also presents the experiences of shipping companies and Seafarers’ Missions after severe mental stress has occurred on board.

## Methods

An examiner accompanied 22 sea voyages (16 small container ships on exclusively coastal voyages and 6 large container ships with worldwide shipping routes) and interviewed the entire crew about heavy mental loads or potentially traumatic events. Of the 365 crew members, 323 seafarers took part in this study (participation rate 88.5%). Participation in the study was completely voluntary and the data collection was pseudonymised. All participants gave their written informed consent before taking part in this study. The study was approved by the Ethics Committee of the Hamburg Medical Association (no PV4395).

The study sample consisted of 156 Europeans (48.3%) and 167 non-Europeans (51.7%). The average age of the exclusively male crew members was 38.2 years (± 11.7 years). For professional stratification, a further distinction was made between the 201 ratings (62.2%) and the 122 officers (37.8%).

The recording of the experienced extreme stress and possible psychological after-effects for the crew members after a potentially traumatic event was carried out through a standardized interview. The latter examination procedure based on previously conducted qualitative interviews [7, 8] as well as a subsequent testing in three unpublished pilot studies. The estimation of possible psychological after-effects, according to the ICD-10 V (F43.1), refers to characteristics such as unintentional repeated experience of the event, imposing memories or frequent dreams of the stress situation (see Table 1). The standardized interview was conducted by a psychologist experienced in seafaring who was able to control the comprehensibility of the items for the multicultural crew members.

**Table 1:** Consequences of strong traumatic experiences on board

	Total sample (323)	Europeans (156)	Non-Europeans (167)	Ratings (201)	Officers (122)
Consequences of a shipwreck or serious personal injury (*n* = 116); *n* (%^a^)					
Often thinking about it, also unintentionally	***45 (38.8%)***	27 (48.2%)	18 (30.0%)*	24 (30.8%)	21 (56.8%)**
Being reminded of it through certain sounds or smells	***34 (29.3%)***	21 (37.5%)	13 (21.7%)*	20 (25.6%)	14 (37.8%)
Dreaming about it	***18 (15.5%)***	8 (14.3%)	10 (16.7%)	11 (14.1%)	7 (18.9%)
Consequences of a serious robbery or piracy (*n* = 55); *n* (%^a^)					
Often thinking about it, also unintentionally	***16 (29.1%)***	7 (22.6%)	9 (37.5%)*	10 (34.5%)	6 (23.1%)
Being reminded of it through certain sounds or smells	***21 (38.2%)***	10 (32.3%)	11 (45.8%)*	14 (48.3%)	7 (26.9%)
Dreaming about it	***5 (9.1%)***	3 (9.7%)	2 (8.3%)	3 (10.3%)	2 (7.7%)

Chi^2^-test: *p < 0.05 and > 0.01; **p < 0.01 and > 0.001

^a^ related to the seafarers who had experienced a corresponding event

In addition, interviews were carried out at 12 German shipping companies: with the owners of the companies (5 times), the managing directors (4 times) or the fleet/crewing managers (2 or 1 time/s). The randomly selected 12 German shipping companies (of a total of 108 German companies) owned between 1 and 72 container ships and were considered as representative for the German container ship fleet in terms of crew structure and underlying safety standards.

Furthermore, a psychologist conducted interviews with the leaders (a total of 4 theologians and 4 deacons) in 8 stations of the German Seafarers’ Mission.

The surveys of the shipping companies and Seafarers’ Missions were based on guided interviews (semi-standardized interviews). A flexible set of questions was compiled during the development of the interview guideline relying on the results of a self-imposed survey of seafarers [9]. The evaluation was qualitatively based on the coding procedure of Jamshed [8] as well as Kvale and Brinkmann [10]; during the coding, a text passage or records could be assigned to an interview code, i.e. keywords or terms. In a synopsis, the results of the interviews were compiled separately, supplemented and compared with each other independently by two scientists.

### Statistical analysis

Data analysis was performed with SPSS for Windows (version 20.0, SPSS GmbH Software, Munich, Germany). Continuous variables were presented as mean (± standard deviation (SD). The Pearson Chi-square test was applied to compare frequencies between groups. All indicated *p*-values were two-sided, and a *p*-value of < 0.05 was considered as statistically significant.

In order to estimate the number of deaths in the study sample, the fatal events in relation to the sum of the seafarer years were calculated in the three-year surveyed period and converted to 100,000 seafarer years [11].

## Results

Of the 323 study participants, 116 seafarers (35.9%) had experienced ship wrecks or serious accidents. As consequences (according to ICD-10 V (F43.1)), 38.8% of these crew members stated often having to unintentionally think about the smells, 29.3% expressed that they were reminded of the event through certain sounds or smells and 15.5% that they dreamt about it. Officers were significantly more likely than ratings (*p* = 0.003) to often unintentionally think about the shipwreck or the serious accident on board (Table 1).

Fifty-five seafarers (17.0%) experienced a serious robbery or piracy with the after effects of often unintentionally thinking about it (29.1%), being reminded of it through certain sounds or scents (38.2%) and dreaming of the event (9.1%).

One hundred twenty-six seafarers (39.0%) had experiences with stowaways, more frequently officers than ratings (46.2% vs. 35.4%; *p* = 0.05). In one case, 28 stowaways were discovered in a container and difficulties were encountered in handing them over to the port authorities. After adjusting for age, all observed associations of the cultural and occupational characteristics to the consequences of the traumatic events remained significant.

Of the 126 seafarers with experience with stowaways on board, 50.0% regarded them as a high burden. The main aspects of this burden were problems with the port authorities (58.7%), internal conflicts in treating the stowaways with human dignity, on the one hand, and, on the other hand, having to comply with legal requirements (40.0%), threats to crew (38.1%), high administrative requirements (36.5%), uncertainty about how to behave (34.9%) and physical violence by the stowaways (25.4%).

In particular, non-European seafarers (51.5% vs. 23.3%, *p* = 0.021) and ratings (52.9% vs. 20.7%, *p* = 0.009) had experienced a threat to the crew by stowaways. This was especially the case for deck crews. Physical violence had tended to be more common among non-European seafarers (and thus many ratings) than among European crew members (27.3% vs. 23.3%, not significant).

### Interviews with shipping companies

Based on the crew structure and the size of the 12 shipping companies examined, a representativeness of this study sample was assumed. According to the interviews with these shipping companies, there were 14 deaths on their container ships in the last 3 years. In 6 cases, the causes of these deaths were heart attacks or serious cancers, in 3 cases very serious accidents and 2 suicides; 3 causes of death are unknown. In the 3-year period surveyed, a median of 840 seafarer years (between 60 and 8545 seafarer years) was recorded. The 14 deaths represent a crude mortality rate of 78.4 per 100,000 seafarer years.

In cases of strong traumatic experiences on board, 3 shipping companies would directly contact the Seafarers’ Mission for initial psychological support. Especially within a Filipino crew, the death of a crew member requires first and foremost pastoral support.

If necessary after traumatic experiences on board, some shipping companies would contact the agent (twice), the insurer (twice), the telemedical assistant service (twice) or the maritime rescue centre (twice) for mental health support and also try to replace affected crew members. By hiring out employees, one shipping company made it possible for a smaller shipping company to soon replace a traumatized seafarer.

Three shipping companies saw no need for psychological support after extreme stress on board and assessed that this help is the responsibility of the crewing agency alone.

### Interviews with seafarers’ missions

According to the interviews with the Seafarers’ Missions, these organizations are particularly active in emergency counselling in the form of crisis intervention. A focal point in the case of a death on board is religious assistance and devotion. After traumatic experiences, the strongly religiously bound Filipino seafarers would try to get in touch with a Catholic priest. Through on-board visits, if possible with longer stay (as it is, for example, possible in the passage through the Suez Canal) one-on-one discussions, group discussions, but also devotions were carried out. In cases where no direct personal help in the form of religious assistance or other help is available on the spot, initial contact takes place for about half an hour via internet and skype. A communication of information for a personal debriefing will then be carried out by a native speaker in the ship’s next port of call. A shipping company interested in pastoral-psychological support can facilitate board access by seeing to the necessary formalities, such as visas in advance. The staff of the Seafarers’ Missions is trained for dealing with the tasks of crisis intervention in the English language, which is common in seafaring.

## Discussion

The present study revealed that uncontrolled and unintentional thoughts after shipwrecks and personal accidents occurred significantly more frequently among European crew members and especially among officers. Possibly, the officers were more mentally involved in these events because of their leadership responsibility.

The Filipino crew with their socio-centered bonds and religious-magical values are likely to be particularly troubled if one of their group members dies or goes overboard and is severely injured. In such cases, feelings of “supernatural” threats arise among the affected Filipino and often result in excessive tendencies to want to escape from the scene of the accident [12, 13]. On the other hand, this religiousness in connection with religious activities is an important resource for crisis management. According to statements by some shipping company representatives, stabilization among the Filipino occupational group can be achieved by priestly actions on board [14, 15].

Considering potential traumatic events, Maercker and Mehr [15] distinguish between man-made disasters, i.e. caused by humans (e.g. piracy etc.) and accidental disasters (such as catastrophes, accidents, etc.). It is often assumed that people from East Asian cultures are more often affected mentally by man-made disasters due to their stronger collectivism [16, 17]. The observed more frequent psychic consequences of a perceived piracy threat among non-European seafarers could be an indication of this. In this context, there is a need for further research, particularly as there are no/few secured research results on this topic [18]. Regardless of the cultural background, Resnick et al. [19] described in a study that human-induced psychic traumas, for example crimes, have a more serious impact than “non-interpersonal disasters”, such as natural catastrophes.

According to Terheggen et al. [20], there are cultural differences in the response to potentially traumatic events. They observed that for religiously bound people from East Asian cultures the destruction of religious symbols is often experienced as even more traumatic than their own mortal danger. With regard to the cultural differences in Posttraumatic Stress Disorders, Hinton and Lewis-Fernandez [21] found that in socio-centric societies, somatic symptoms after traumatic exposures were more common.

According to Grover and Ghosh [22] and Bagayogo et al. [23], the tendency for increased somatization of mental disorders is to be seen in the different culture-related way in which mental disorders and diseases are regarded. Accordingly, mental disorders, especially in the Asian culture, are seen as a weakness and are often even stigmatized. That is why mental disorders often manifest in somatic forms such as headaches, back pain etc.

Both physical and mental disorders must also be seen in connection with the socio-economic situation and the family relationship. Being helpless because of mental stress reactions and disorders means a loss of status and face in the family [24, 25]. In addition, the unsecured economic status leads to the fear that a mental disorder could result in a replacement and thus in a risk to the workplace, leaving them unable to support their families.

Currently, extreme stress is also possible during the rescue of refugees on the high seas. Due to escalating political unrest, the number of refugees fleeing across the Mediterranean has increased in recent years. In the past, merchant ships have been repeatedly involved in large-scale rescue operations of refugees stranded on the high seas. Often the ship’s crews are overwhelmed because of their low personnel levels and, above all, their lack of experience with large-scale rescue operations on the high seas [26]. The sometimes dramatic rescue experiences, especially dealing with the injured and the sick, as well as the confrontation with drowned people, also represent a considerable psychological burden for the crews on merchant ships. Therefore, crisis intervention is of great importance, i.e. psychological first aid for seamen with strong stress responses in the context of post-processing. According to Scuri et al. (2019) [27] rescue workers should also be prepared for emergencies situations. They observed that resilience and coping strategies within the framework of self-preservation are important personnel prerequisites for dealing with emergency situations.

Trauma pilots from the Berufsgenossenschaft Verkehr provide information about possible competent support, treatment options and suitable therapists after a potentially traumatic event on board. Basically, initial psychosocial help after a potentially traumatic event should be given by a neutral, trained person with experience in seafaring who is not involved in the hierarchical structure of a ship’s crew. As, in the closed social system of a ship with a small crew, practically every crew member is indirectly affected by a potentially traumatic event, initial psychosocial help by a suitably trained peer from the crew is not recommended. But this is not a realistic scenario, anyway. For Filipino crews with their strong religious ties, pastoral support should be sought through the Seafarers’ Mission.

As far as possible, however, every seafarer, first and foremost the superiors, should have a basic knowledge of the very serious psychological stress caused by shipwrecks, accidents, external violence, etc. Psychoeducation needs to address possible physical and psychological reactions to a potentially traumatic event. It should be noted that the physical and psychological symptoms are normal responses to such an event. This perception can be a relief to the affected crew members. Simple codes of conduct combined with opportunities for short-term relaxation can be very helpful and provide appropriate guidance and support. It is important for anyone affected to gradually find their way back into the structure of everyday life.

Psychoeducation for ratings should convey information in simple and understandable words - possibly in the native language - by using appropriate media, such as DVDs. This information should also take cultural perception and experience of extreme mental stress, especially among Asian crew members, into consideration. For European crew members, psychosocial first aid seems to be in the to the experiences of the Seafarers’ Mission, Western Europeans are more planning-oriented and prefer to plan in advance. Asians, on the other hand, “let things happen and only then decide how to react”. This corresponds to Frey and Jonas’ concept of cognized control [28], i.e. in seemingly confusing, unclear and uncontrollable situations it is only possible to think clearly when action is absolutely necessary. This behavioural tendency among seafarers from Asian cultures should be considered when preparing for extreme stress situations. In contrast, Europeans are more inclined to mentally engage with the situation and intervene in actions. According to Antonovsky [29] and Sones et al. [30], having a sense of predictability, control and of meeting competence expectations has a positive effect on how individuals cope with extreme burdens.

## Conclusions

Leaders should be empowered to recognize signs of psychological overburdening, instability, and shock in their crew members and to provide assistance wherever possible. Psychoeducation should therefore be established as a preventive measure for extreme mental stress situations. Nevertheless, it is not possible to prepare for all crisis situations that can lead to extreme psychological stress. However, it is important to attend to immediate stress reactions and to maintain the control belief. Ideally, according to Davies [31] and Meichenbaum [32], the seafarers’ partners should be included in certain training sessions as they are affected by the psychological stress reactions of their partners and may be able to give them support. However, normally this does not seem feasible as seafarers come from countries throughout the world.

## Data Availability

The datasets used and/or analysed during the current study are available from the corresponding author on reasonable request.

## Abbreviations

BSU: Federal Bureau of Maritime Casualty Investigation
PTSD: Posttraumatic Stress Disorder
SD: Standard Deviation
